# Modulation of the proteoglycan receptor PTPσ promotes white matter integrity and functional recovery after intracerebral hemorrhage stroke in mice

**DOI:** 10.1186/s12974-022-02561-4

**Published:** 2022-08-18

**Authors:** Min Yao, Jie Fang, Jiewei Li, Anson Cho Kiu Ng, Jiaxin Liu, Gilberto Ka Kit Leung, Fanglai Song, Jian Zhang, Chunqi Chang

**Affiliations:** 1 School of Pharmaceutical Sciences, Health Science Centre, Shenzhen University, Shenzhen, 518060 China; 2 School of Biomedical Engineering, Health Science Center, Shenzhen University, Shenzhen, 518060 China; 3 Department of Surgery, LKS Faculty of Medicine, The University of Hong Kong, Hong Kong, China

**Keywords:** Intracerebral hemorrhage, PTPσ, ISP, Neural circuits integrity, Neuroinflammation

## Abstract

**Background:**

Intracerebral hemorrhage (ICH) is associated with high morbidity and mortality rates. However, extant investigations have mainly focused on gray matter injury within the primary injury site after ICH rather than on white matter (WM) injury in the brain and spinal cord. This focus partly accounts for the diminished therapeutic discovery. Recent evidence suggests that chondroitin sulphate proteoglycans (CSPG), which can bind to the neural transmembrane protein tyrosine phosphatase-sigma (PTPσ), may facilitate axonal regrowth and remyelination by ameliorating neuroinflammation.

**Methods:**

A clinically relevant ICH model was established using adult C57BL/6 mice. The mice were then treated systemically with intracellular sigma peptide (ISP), which specifically targets PTPσ. Sensorimotor function was assessed by various behavioral tests and electrophysiological assessment. Western blot was used to verify the expression levels of Iba-1 and different inflammatory cytokines. The morphology of white matter tracts of brain and spinal cord was evaluated by immunofluorescence staining and transmission electron microscopy (TEM). Adeno-associated virus (AAV) 2/9 injection was used to assess the ipsilateral axonal compensation after injury. Parallel in vitro studies on the effects of CSPG interference on oligodendrocyte–DRG neuron co-culture explored the molecular mechanism through which ISP treatment promoted myelination capability.

**Results:**

ISP, by targeting PTPσ, improved WM integrity and sensorimotor recovery via immunomodulation. In addition, ISP administration significantly decreased WM injury in the peri-hematomal region as well as cervical spinal cord, enhanced axonal myelination and facilitated neurological restoration, including electrophysiologically assessed sensorimotor functions. Parallel in vitro studies showed that inhibition of PTPσ by ISP fosters myelination by modulating the Erk/CREB signaling pathway.

**Conclusions:**

Our findings revealed for the first time that manipulation of PTPσ signaling by ISP can promote prolonged neurological recovery by restoration of the integrity of neural circuits in the CNS through modulation of Erk/CREB signaling pathway.

**Supplementary Information:**

The online version contains supplementary material available at 10.1186/s12974-022-02561-4.

## Background

Intracerebral hemorrhage (ICH) is a cerebrovascular disease that has devastating effects on health. The global prevalence of stroke in 2019 was 101.5 million people, of which 20.7 million involved ICH [1]. In general, brain injury after ICH involves primary brain damage resulting from hematoma or oedema, followed by secondary brain damage resulting from activation of the immune system, inducing grey and white matter (WM) injury [2, 3]. ICH always involves the internal capsule, which is the WM region composed of multiple fiber tract groups, including the cortical spinal tract (CST), connecting cerebral cortex, and spinal cord. The resultant axonal tracts blocking information transmission could induce sensorimotor deficits, including hemiplegia and hemidysesthesia [4]. Various therapeutic studies of white matter, including CST after brain injury or stroke, have been investigated repeatedly in the brain and brainstem; however, the presence of extensive degeneration into the spinal cord has rarely been mentioned [5–7].

After stroke, the upregulation of the expression of various extracellular matrix molecules, such as chondroitin sulphate proteoglycans (CSPG), which consist of chains of sulfated glycosaminoglycans (GAGs), is a key biomarker of the scarring process [8, 9]. The neural transmembrane protein tyrosine phosphatase-sigma (PTPσ) has been identified as a receptor for the inhibitory actions of CSPG [10, 11]. Recent research has demonstrated that CSPG blocks axonal regrowth by targeting PTPσ, prevents oligodendrocytes progenitor cell (OPC) maturation and remyelination after spinal cord injury (SCI) and multiple sclerosis (MS), and also negatively modulates the survival, proliferation, growth, and attachment of neural precursor cells through the Rho/ROCK pathways after SCI [12–14].

During this inflammatory process of ICH, the role of microglia is indispensable [15]. In the acute phase, activation of microglia to an M1-like (proinflammatory) phenotype occurs and produces proinflammatory cytokines, proteases, and reactive oxygen species (ROS). Existing studies show that the secretion of IL-1β, IL-6, and TNF increases in the first few days in collagenase- and blood-induced rodent models of ICH. These cytokines mainly exert destructive effects in the brain [16, 17]. M2-like (anti-inflammatory) microglial responses occur in the subacute and chronic phase, and secrete anti-inflammatory cytokines or enzymes, including IL-4, IL-10, and Arginase-1 (Arg-1), to reduce lesion volume and neurological deficits following ICH [18]. IL-10 expression enhances phagocytosis by monocytes and also induces production of SOCS1 and SOCS3 to restrain proinflammatory cytokine production through STAT3 in macrophages [19]. Arg-1 is a kind of cytosolic enzyme which is expressed by M2b and M2c subtypes of M2 microglia, shown in the promotion of neurological function recovery that is associated with IL-4 and IL-13 [15]. In addition, recent research demonstrates that Arg-1 reduced neurovascular degeneration after ischemia reperfusion injury [20]. Furthermore, in secondary brain damage involving axonal tract disorder following ICH, microglia also play a key role [15]. Current evidence suggests that digestion of upregulated CSPG by ChABC improves immune responses and reduces pathology after SCI [21, 22].

Intracellular sigma peptide (ISP) has been designed previously. This membrane-permeable peptide mimetic of the PTPσ wedge domain can competitively bind PTPσ [12]. Recent studies have shown that modulation of PTPσ alleviates axonal inhibition induced by CSPGs and enhances functional recovery in spinal cord contusion injury, spinal dorsal root crush injuries, and ischemic heart attack [12, 23, 24]. However, the role of targeting PTPσ in changes of immune responses in the brain and subsequent microstructural alterations of WM tracts in the spinal cord after ICH are not well understood. Moreover, clinical surgical hematoma aspiration does not significantly improve prognosis yielded by retrospective methods, emphasizing the need to investigate new strategies [25, 26].

In the present study, use of ISP in a clinically relevant model of ICH in mice has revealed an immunomodulatory role, whereby targeted PTPσ receptors affects WM integrity and sensorimotor functional recovery. We demonstrate that after ICH, systemic treatment with ISP decreases microglia activation and promotes production of anti-inflammatory cytokines, including IL-10 and Arg-1, which are secreted by M2 microglia. ISP decreased the production of proinflammatory cytokines including TNF-α and IL-1β, which are secreted by M1 microglia. In addition, ISP administration ameliorates WM injury and facilitates axonal myelination and neurological restoration, including sensorimotor functions based on electrophysiological tests. Our parallel in vitro studies on co-cultures of oligodendrocytes and DRG neurons with CSPG interference showed that inhibition of PTPσ by ISP enhances myelination by modulating the Erk/CREB signaling pathway. In addition, we explored potential molecular pathways which significantly participate in inflammatory and neurogenesis processes. Taken together, our findings may have significant clinical relevance in therapeutic approaches developed for ICH.

## Methods

### Animals

All animal care and animal procedures were approved by the Animal Ethical and Welfare Committee of Shenzhen University. Animals were purchased from Guangdong Medical Laboratory Animal Center. For in vivo experiments, wild-type C57BL6/J male mice (12–14 weeks), and for in vitro studies, C57BL6/J mice pups were used. The mice were housed in the Animal Research Center in Shenzhen University, maintained with 12-h/12-h light–dark cycle with ad libitum access to chow and water. The animal holding areas were under constant monitoring.

### Experimental intracerebral hemorrhage (ICH) model and treatments

**Fig. 1:**
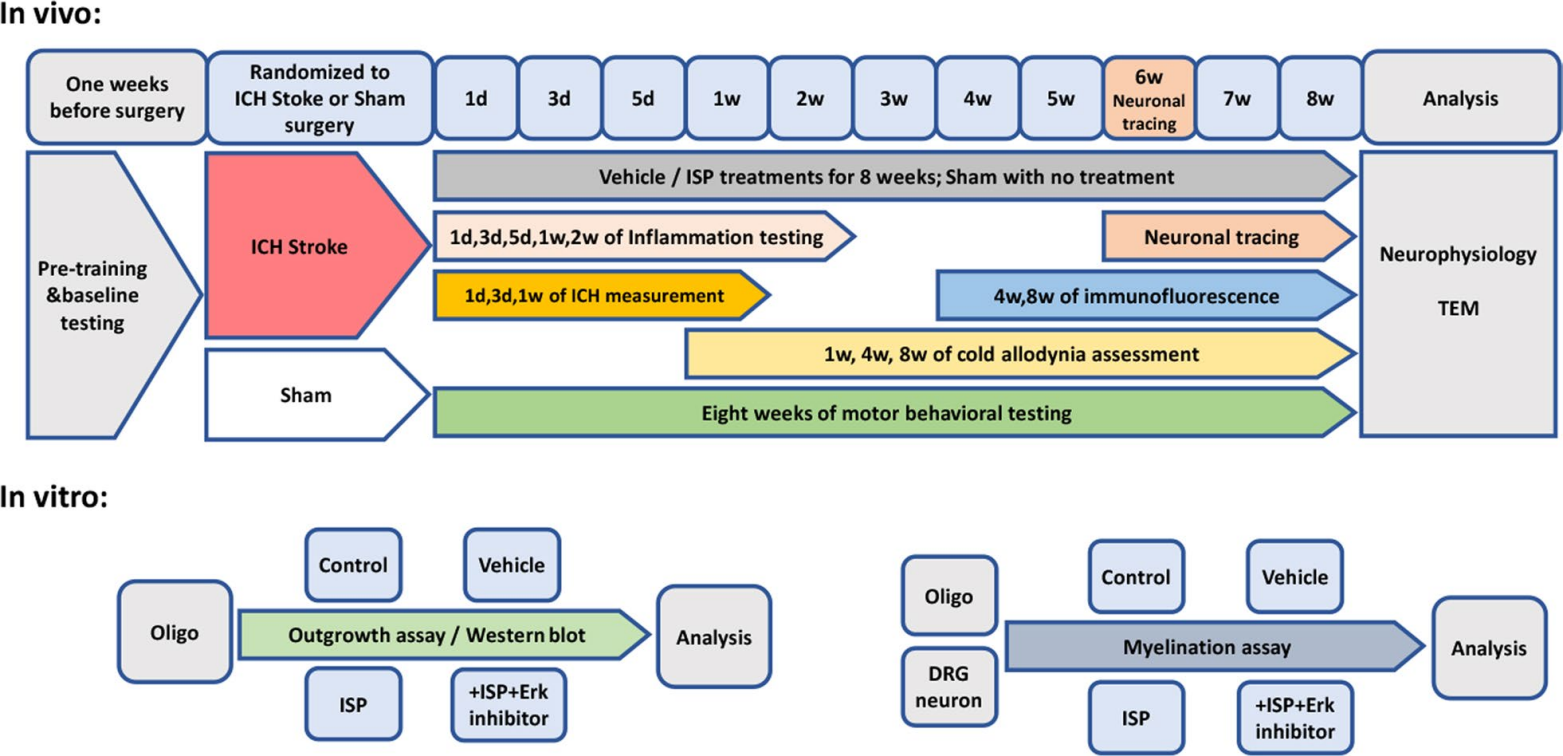
Summary of the experimental procedures, timepoints, and different treatment groups from the in vivo and in vitro experiments

Anesthesia was induced with 4% isoflurane and maintained with 2% isoflurane. The mice were placed in stereotactic frame (RWD Life Science Co., Shenzhen, China). Intracerebral hemorrhage was induced by intracranial injection of type IV collagenase (Sigma-Aldrich, St. Louis, MI, United States) into the right corpus striatum (coordinates from bregma: 2.0 mm lateral, 0.5 mm posterior and 3.5 mm ventral). Then ICH was induced by a slow injection of 0.08U type IV collagenase in 0.5 µL saline at a rate of 0.05 µL/min [27]. ISP (20 µg/day, 100µL, synthesized by CSBio) for treatment group, or Vehicle (5% DMSO in saline, 100 µL) for Vehicle group, were given daily intraperitoneal injections at 12 h post-ICH model built for different experimental timepoints (Fig. 1). The dose of ISP was referred by our pre-experiment and previous study [13].

### Functional behavioral assessments

All injured mice were tested at least once prior to injury to obtain baseline responses. From the first week post-ICH injury, the tests were conducted three times. Three different assessments were involved: ladder rung walking, cylinder test, and acetone test.

1. Ladder rung walking: this assessment was used to measure (1) the foot placement error ratio and (2) the foot score on the affected forelimb during walking test [28]. Mice were placed into the apparatus consisted of 1 m long and 19 cm height. Video recording of body and paw positions was taken. Error ratio ranged from 0 to 2 points, and foot score rating system ranged from 0 points (total miss) to 6 points (correct placement). The score and error ratio were calculated as: (sum of foot score per step) / (number of foot step); (sum of error per step) / (number of foot step), respectively.
2. Cylinder test: this assessment was used to detect mice forelimb impairments with unilateral lesions when rearing to explore the environment [29, 30]. An angled mirror was placed behind the cylinder so that the movement could be identified when the animal turned away. The forelimb score was recorded as contralateral or ipsilateral independent placement of the forelimb to touch the wall. The “both” score was recorded as both contralateral and ipsilateral placement of the forelimbs to touch the wall during a full rearing. Scores were obtained from 10 full rears. The asymmetry of contralateral forelimb usage was calculated as: (contralateral forelimb use + 1/2 bilateral forelimb use) / (contralateral forelimb use + ipsilateral forelimb use + bilateral forelimb use).
3. Cold allodynia test. The assessment was processed with standard methods [31]. Mice were placed in a transparent cylinder with a mesh floor. A drop (20 µl) of 4 °C acetone was applied to the center of the forepaw. The score of animal’s responses was recorded within 40 s following the scale: 0, no response; 1, brisk withdrawal or flick of the paw; 2, repeated flicking of the paw; 3, repeated flicking of the hind paw and licking of the paw. The test was repeated alternately thrice to each paw. The score is the summation of the thrice grades of each paw.

### Neurophysiology

Mice were involved to assess the somatosensory evoked potentials (SEP). At 8-week post-ICH, 38 mice (12 mice for Sham group; 12 mice for Vehicle group; 14 mice for ISP group) were anesthetized with 4% isoflurane and maintained with 2% isoflurane. The mice were kept with heating pad and placed in stereotactic frame. The skull was exposed by skin dissection with removal of a bone with diagram around 1 mm on the right side. Then the tetrode recording electrode (Bio-signal technologies, China) was gently lowered through the right-side S1FL perpendicularly (coordinates from bregma: 1.8 mm lateral, 0.7 mm anterior and up to a maximum depth of 1 mm ventral from pia). Stimulating electrode was inserted subcutaneously on the left side forelimb around the medium nerve with 0.2ma, 1 ms, 0.897–1.270 s interval. Responses were recorded (Recorder: BLACKROCK; Logging software: CerePlexDirect) simultaneously. SSEP signals was sampled at 10 kHz and bandpass filtered between 1 and 90 Hz. Before averaging the SSEP waveforms, the “baseline” value was removed of each trial by subtracting the mean value of 200 ms samples before stimulus onset. We mainly measured three components of the averaged SSEP waveform, including the first two positive peak (P1 and P2) and the first negative peak (N1). The amplitudes and latencies of three components was compared between different experiments groups. Moreover, the time–frequency distribution (TFD) was also calculated by the short-time Fourier Transform (STFT) method of the averaged SSEP.

matlab version: 2019a.

EEGLAB version: v2020.0

short-time Fourier Transform (STFT) formular:

https://www.mathworks.com/help/signal/ref/stft.html?s_tid=srchtitle_STFT_1

Median filter:

https://www.mathworks.com/help/signal/ref/medfilt1.html

Bandpass filter: (eeglab).

https://sccn.ucsd.edu/pipermail/eeglablist/2016/011854.html

### Anterograde neuronal tracing and axonal sprouting quantification

The mice were anesthetized with 4% isoflurane and maintained with 2% isoflurane After placing on the stereotaxic frame, skull was exposed and holes were drilled at the right side primary motor cortex (M1) for the rAAV–CaMKIIa–mCherry (BrainVTA) viral injection using syringe pump controller (Harvard Apparatus) (0.6 µl total volume; 40 nl/min rate; coordinates from bregma: 2 mm lateral, 1.75 mm anterior and 0.8 mm; 2 mm lateral, 1.25 mm anterior and 0.8 mm ventral from pia). Mice were sacrificed after 2 weeks; brain and cervical spinal cord were harvested to examined the axonal sprouting. Cryostat sections were performed with brain and cervical spinal cord at 30 µm and 25 µm thickness. The number of midline crossing fluorescent positive fibers (from contralateral to ipsilateral side) was manually counted by a blinded experimenter.

### Immunofluorescence and image analyses

The brain and the spinal cord were fixed by 4% PFA perfusion and dehydrated in sucrose for cryostat sectioning. Transverse sections in 30 µm thickness for the brain and 25 µm thickness for the spinal cord were collected for immunofluorescent staining. Primary antibodies were used: rabbit anti-MBP (1:100; Cell Signaling technology), Mouse anti-SMI-32 (1:100; BioLegend). Sections were then incubated in Alexa Fluro-conjugated secondary antibodies (1:500, Invitrogen) for 2 h in room temperature and mounted in antifade Mounting Medium (Beyotime). Images were photographed and analyzed by ZEISS LSM880 fluorescence microscope.

### Transmission electron microscopy (TEM)

Preparation of sections and analysis were described previously [23, 27]. After 8 weeks of different treatments, 2–3 mice per group were deeply anesthetized by pentobarbital sodium (200 mg/kg, intraperitoneally). The cervical segment of the spinal cord was harvested and fixed overnight with a mixture of 2% PFA and 2.5% glutaraldehyde in 0.1 M PB, followed by overnight fixation with 1% osmium tetroxide. After progressive dehydrated in graded ethanol, samples were embedded in epoxy resin. Ultrathin (100 nm) transverse sections sectioned with an ultramicrotome (UC6, Leica, Wetzlar, Germany) for TEM analysis to delineate the phenotypic changes of CST of the cervical spinal cord. Images (1200 × , 5200 ×) were viewed and taken under a TEM (CM100 Transmission Electron Microscope, Phiolips, Eindhoven, Netherlands).

### In vitro cell culture and immunofluorescence

Oligodendrocytes precursor cell (OPC) were primary cultured [32]. OPCs were harvested from the cerebral cortex of neonatal C57BL6/J mice. The cerebral cortex was diced and digested in 0.25% trypsin (Invitrogen) with 0.2 mg/ml DNase I (Invitrogen) at 37 °C for around 15 min. After tissue dispersed, cells were incubated in DMEM20s [Neurobasal medium (Invitrogen) + 4 mM Glutamax (Invitrogen) + 1 mM Sodium Pyruvate (Thermo fisher Scientific) + 10% FBS (Invitrogen) + 1 × P/S (Invitrogen)] in flask. The medium was changed every other 2 days. Around 10 days, OPCs were obtained by shaken flask at 200 r.p.m for 12 h at 37 °C and seeding onto flask coated with 50 µg/ml PDL-o in OPC proliferation medium [Neurobasal medium (Invitrogen) + 1 × B27 (Thermo fisher Scientific) + 1 × Glutamax (Invitrogen) + 10 ng/ml bFGF (Sigma) + 10 ng/ml PDGF-AA (Sigma)]. For the measurement of oligodendrocytes outgrowth, OPCs were cultured in the Differentiation medium [Neurobasal medium (Invitrogen) + 1 × B27 (Thermo fisher Scientific) + 1 × Glutamax (Invitrogen) + 5 µg/ml NAC (Sigma) + 15 nM T3 (Sigma) + 10 ng/ml CNTF (Peprotech)]. OPC differentiation assay contained four groups: (1) Con (no interference); (2) agg: aggrecan (25 μg/ml in PBS, Sigma) for 3 h; (3) agg + ISP (2.5 µM ISP followed by aggrecan); (4) agg + ISP + Erk inhibitor (3 and subsequent 10 µM U0126 (Sigma) for 1 h).

Dorsal root ganglion neuron (DRG) was also primary cultured for the co-culture with OPC [33]. Dissociated DRG cells from neonatal rats were cultured as described previously [34]. Before OPCs seeded onto neuronal coverslips, several pre-treatments were processed for OPCs as described above.

After the cells fixed and blocked, the in vitro immunofluorescence analysis was performed. Primary antibodies were used: rabbit anti-NF200 (1:400; Sigma;), Mouse anti-MBP (1:50; Millipore). After washed in PBS, the coverslips were then incubated in Alexa Fluro-conjugated secondary antibodies (1:500, Invitrogen) for 2 h in room temperature and mounted in antifade Mounting Medium (Beyotime). Images were photographed and analyzed by ZEISS LSM780 fluorescence microscope.

### Western blot assay

For the western blotting, brain tissue or cultured cells were grinded and homogenized in RIPA (Beyotime) and protease inhibitor (Beyotime). Protein extracted from perilesional tissue was analyzed by western blotting. The penumbra around the hematoma in our study was 0.5 mm thick. Perilesional tissue was extracted randomly from that region. For the control group, brain tissue was harvested from the sham group mice at the same location, where the tissue was dissected from the ICH mice. The same amount of total loading proteins (15–50 g) was separated by SDS/PAGE according to standard protocols and transferred to nitrocellulose membranes. Primary antibodies were used: rabbit anti-Iba-1 (1:1000; Cell Signaling technology), Rabbit anti-TNF-α (1:1000; Abcam), IL-1β (1:500; Abcam), Rat anti-IL-10 (1:1000; Abcam), Rabbit anti-Arginase-1 (1:1000; Cell Signaling technology) for in vivo tissue; Rabbit anti-p-Erk (1:1000; Cell signaling technology), Rabbit anti-p-CREB (1:1000; Cell signaling technology), Rabbit anti-Erk (1:1000; Abcam), rabbit GAPDH (1:2000; Cell Signaling technology). After primary incubation, the membranes were followed incubated within HRP-conjugated anti-rat or anti-rabbit IgG and detected using enhanced ECL detection system (Merck Millipore). Quantification was carried out with ImageJ software (NIH).

### Statistical analyses

Data are represented as the mean ± SD or mean ± SEM. Statistical analyses were performed using Prism 6.0 software. Unpaired Student's t test was used to analyze statistical significance between two groups. One-way ANOVA was used, where more than two groups were compared. Two-way ANOVA was used to compare differences between multiple groups occurring over time. The Bonferroni test was performed following ANOVA analysis. A *P* value of < 0.05 was considered significantly different.

## Results

### ISP treatment improved long-term neurological functional recovery after ICH

**Fig. 2:**
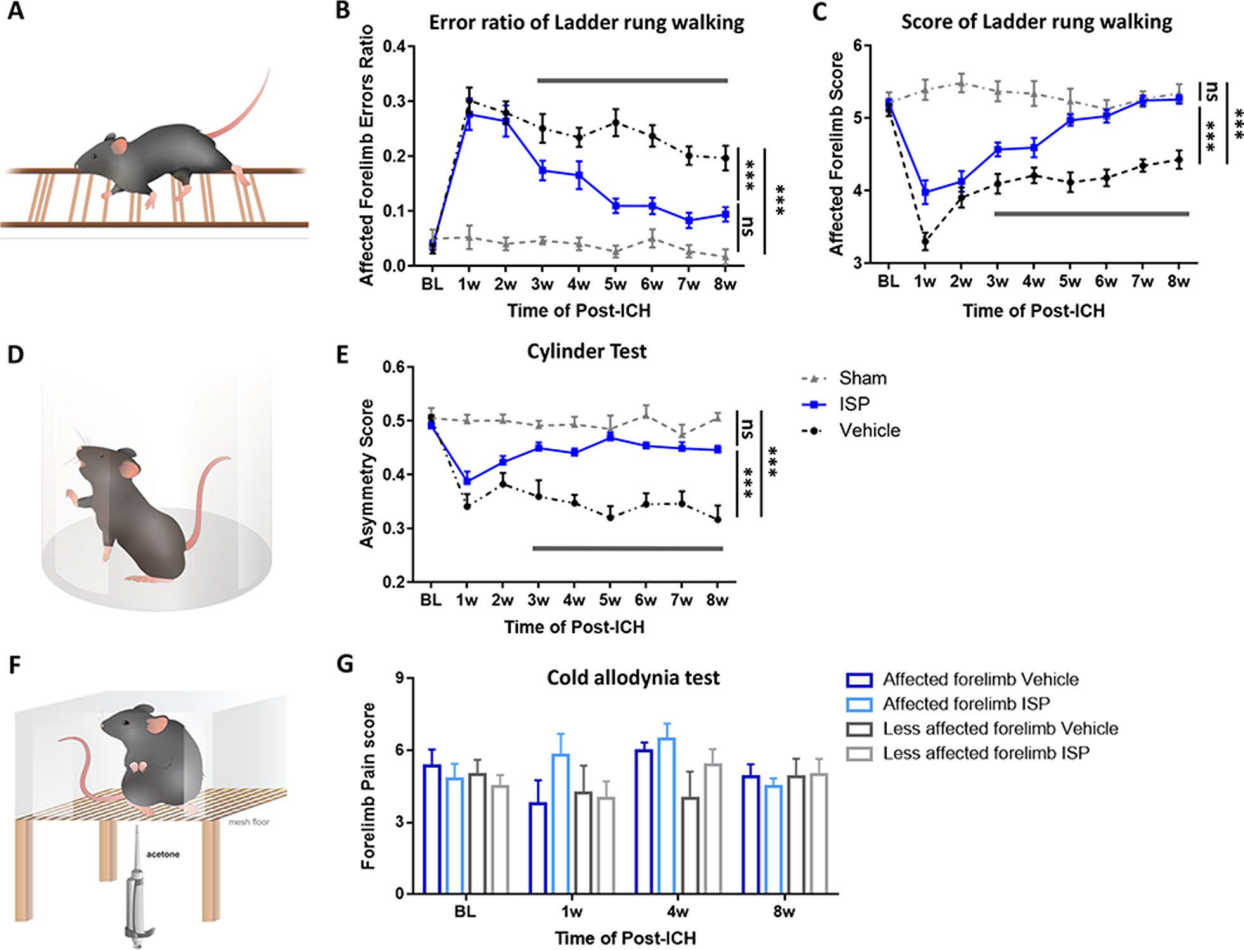
ISP promotes long-term sensory-motor behavioral recovery following ICH. **a** Irregularly spaced horizontal ladder device was used to assess paw placement accuracy during walking. **b**, **c** At week 1 post-ICH, the affected forelimbs of mice in the vehicle and ISP groups had similar error ratios and running scores. However, from week 3 post-ICH onward, ISP-treated mice exhibited progressive and rapid recovery compared to the vehicle group in their error ratios and score. **d** Transparent cylinder was adapted to assess affected forelimb use and asymmetry. **e** Similar reduction was observed in the asymmetry score of affected forelimbs during rearing in both vehicle- and ISP-treated mice 1-week post-ICH. At week 3, ISP-treated mice showed a progressive restoration of the use of the affected forelimb compared to the vehicle treatment group. Vehicle and ISP groups, *n* = 15–17 per group; Sham group, *n* = 5. **f** Mice are placed individually on a mesh floor and applied with acetone. **g** Throughout the experiment, ISP treatment did not induce hypersensitivity of any limbs in all three groups when assessed by the acetone test. Vehicle and ISP groups, *n* = 9–10 per group; Sham group, *n* = 5. Data were analyzed by two-way ANOVA followed by Tukey’s multiple comparisons test, and expressed as mean ± SEM. ***p < 0.001; *ns*, not significant

Hematoma in the striatum caused by ICH stroke induces long-term, unilateral, sensory-motor, behavioral coordination deficits. To examine the sensorimotor coordination functional restoration, mice from different treatment groups were assayed with ladder-rung walking and cylinder tests at different timepoints post-injury (Figs. 1, 2a, d). Behavioral tests were applied once a week from the first until week 8. Following the ICH operation, mice exhibited inordinately high error ratios and low scores for affected forelimbs during ladder-rung walking (Fig. 2b, c) as well as low asymmetry values during rearing in the cylinder test (Fig. 2e). However, ISP treatment significantly improved recovery compared to the vehicle group in both tests from week 3 onward (Fig. 2b, c, e; ladder-rung walking: *F* (2, 34) = 49.37, *p* < 0.0001); score: *F* (2, 34) = 60.92, *p* < 0.0001; cylinder test: *F* (2, 33) = 27.08, *p* < 0.0001). The forepaws of the mice were examined by the cold allodynia test to determine whether any hypersensitivity was induced by systemic ISP treatment (Fig. 2f). Our results did not show any indication that ICH and ISP caused hypersensitivity in both lateral forepaws (Fig. 2 g; *F* (3, 34) = 1.640, *p* = 0.1983). This conclusion is consistent with our previous work in which, following cervical spinal dorsal root crush injury, ISP promoted axonal regrowth geographically and did not induce hyperpathia as assessed in Von Frey and thermal tests. Robust sensory fiber sprouting into the corresponding dorsal horn was observed [23]. Furthermore, though ICH impaired the ipsilateral hindlimb on the ladder-rung test, ISP could improve the response. In addition, there was no significant deficiency in the contralateral side limbs (Additional file 1: Fig. S1; Affected hindlimb errors ratio: *F* (2, 22) = 4.328, *p* = 0.0260; Affected hindlimb score: *F* (2, 22) = 21.47, *p* < 0.0001; less-affected forelimb errors ratio: *F* (2, 22) = 1.961, *p* = 0.1646; less-affected forelimb score: *F* (2, 22) = 2.296, *p* = 0.1243; less-affected hindlimb errors ratio: *F* (2, 22) = 4.509, *p* = 0.0228; less-affected hindlimb score: *F* (2, 22) = 3.150, *p* = 0.0627).

## Inhibition of PTPσ by ISP fosters a beneficial inflammatory response

Previous research showed that degradation of CSPG by ChABC improves anti-inflammatory M2 microglia activation following spinal cord injury (SCI) [21]. Moreover, targeting PTPσ and LAR receptors with ISP and ILP decreases perilesional microglia and macrophage activation after SCI [35]. Here, we aimed to explore the effects of PTPσ suppression on inflammatory responses after ICH.

**Fig. 3:**
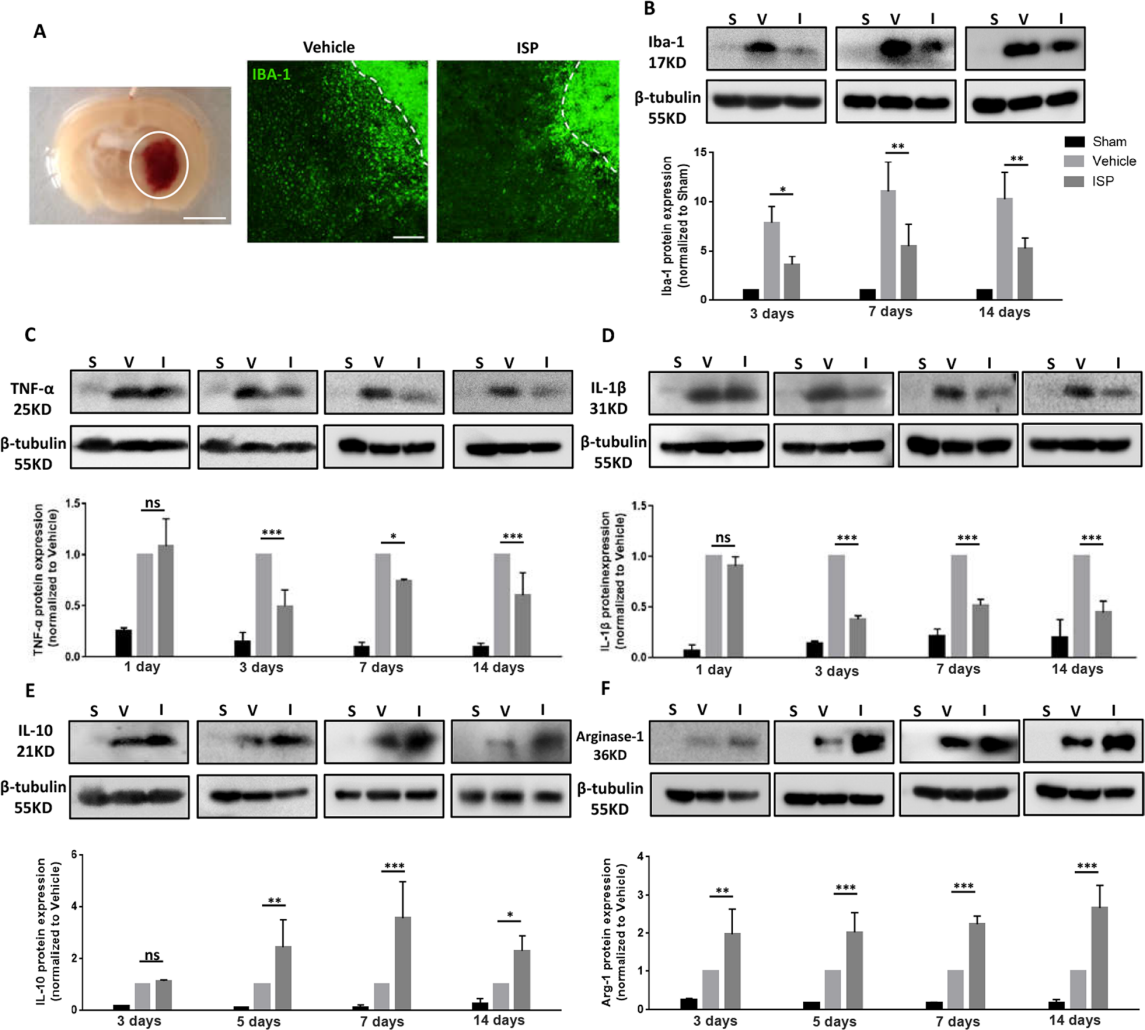
Inhibition of PTPσ by ISP promotes favourable inflammatory responses. **a** Left: protein was extracted from tissue surrounding the ICH shown in the white circle. Scale bar, 2.5 mm. Right: Iba-1 staining around the Hematoma at day 14 post-ICH. Scale bar, 200 µm. **b** Western blot analysis showed that ISP reduced Iba-1 protein expression in the presence of microglia relative to vehicle treatment at days 3, 7, and 14 post-ICH. **c**, **d** Western blot analysis showed a significant reduction in TNF-α and IL-1β protein expression in the ISP group related to the vehicle group at days 1, 3, 7, and 14 post-ICH. Data were normalized to β-tubulin protein expression. **e–f** Western blot analysis showed an increase of IL-10 and Arginase-1 protein expression in the ISP group related to the vehicle group at days 3, 5, 7, and 14 post-ICH. n = 3 per group. Data were analyzed by one-way ANOVA followed by Tukey’s multiple comparisons test, and expressed as mean ± SD. *p < 0.05, **p < 0.01, ***p < 0.001, ns not significant

Protein extracted from perilesional tissue was analyzed by western blotting (Fig. 3a). Microglia are essential immune cells which respond rapidly post-injury. Our results showed that the expression of Iba-1, which is a biomarker for microglia, has a significant and sustained high expression at days 3, 7, and 14 after ICH (Fig. 3b; *F* (2,18) = 59.63; day 3: *p* = 0.0167; day 7: *p* = 0.0022; day 14: *p* = 0.0057). In clinical ICH studies of perihaematomal brain tissue, inflammatory responses set in at early stages, as seen in increased expression of M1-signature (proinflammatory) cytokines TNF-α and IL-1β [36]. The expression changes of different cytokines were analyzed by western blotting, and it was found that pro-inflammatory factors TNF-α (*F* (2,24) = 220.5; day 1: *p* = 0.3938; day 3: *p* < 0.0001; day 7: *p* = 0.0122; day 14: *p* = 0.0002) and IL-1β (*F* (2,24) = 388.1; day 1: *p* < 0.0001; day 3: *p* < 0.0001; day 7: *p* < 0.0001; day 14: *p* < 0.0001) were significantly reduced from the first day after ICH in ISP-treated animals compared to vehicle-treated animals (Fig. 3c, d). By contrast, M2-associated anti-inflammatory markers IL-10 (*F* (2,24) = 49.85; day 3: *p* = 0.9427; day 5: *p* = 0.0099; day 7: *p* < 0.0001; day 14: *p* = 0.0207) and Arginase-1 (*F* (2,24) = 136.5; day 3: *p* = 0.0016; day 5: *p* = 0.0010; day 7: *p* = 0.0001; day 14: *p* < 0.0001) were significantly elevated with ISP administration from day 3 post-ICH onward compared to the vehicle group (Fig. 3e, f). The complete blot results are shown in Additional file 3: Fig S3. In summary, these data demonstrated that ISP enhances the immune responses by increasing the expression of anti-inflammatory and reducing the expression of pro-inflammatory factors.

### Persistent reduction of ICH-induced degeneration by ISP treatment in the striatum and the dorsal CST after ICH

**Fig. 4:**
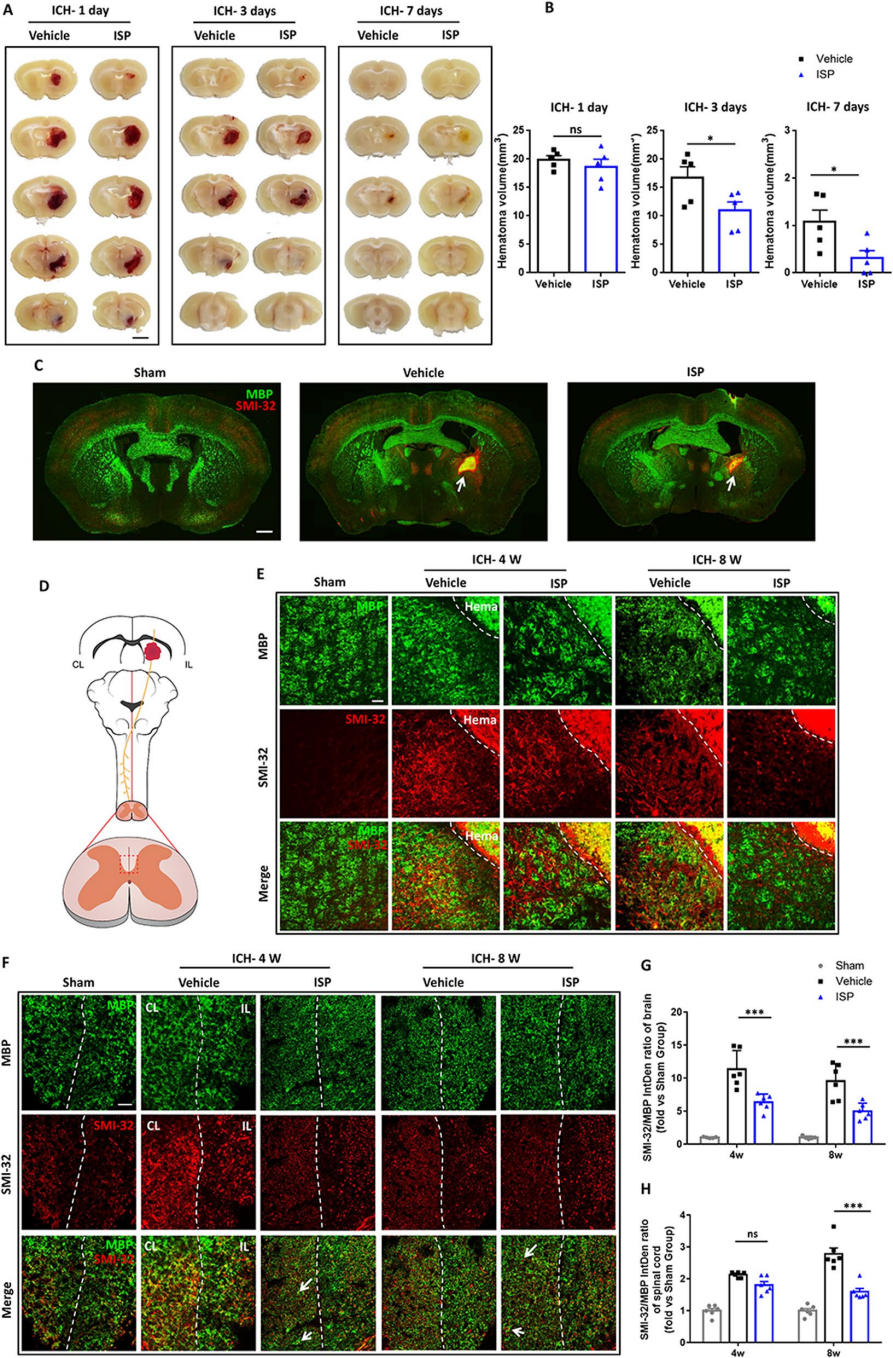
ISP treatment improves long-term brain and cervical spinal cord white matter after ICH. **a** Representative serial coronal brain sections showing the volume of red-hued hematoma in ISP-treated mice were significant smaller than vehicle-treated mice at days 1, 3 and 7 after ICH. Scale bar, 2.5 mm. **b** Quantification of hematoma volumes was based on photographs of consecutive brain sections taken at days 1, 3 and 7 after ICH. **c** Representative coronal sections were stained with MBP and SMI-32 at week 4 post-ICH. White arrow indicates the hematoma in vehicle and ISP groups. Scale bar, 600 µm. **d** Schematic diagram shows that ICH included white matter injury of dorsal spinal cord on the contralateral CST side. **e**, **g** Images of MBP (green) and SMI-32 (red) immunostaining in brain around the hematoma in all groups at weeks 4 and 8 after ICH. Scale bar, 50 µm. Statistical analysis shows a significant reduction in the SMI-32/MBP intensity ratio in the ISP group compared to the vehicle group at weeks 4 and 8. **f**, **h** Images of MBP (green) and SMI-32 (red) immunostaining of contralateral CST in transverse cervical spinal cord sections in three groups at weeks 4 and 8 after ICH. White arrow indicates axons (red) enwrapped by myelin sheath (green). Scale bar, 20 µm. Compared to vehicle treatment, ISP application decreased the SMI-32/MBP intensity ratio of CST at weeks 4 and 8. *n* = 5–6 per group. Data were analyzed by unpaired Student t test between two groups and one-way ANOVA followed by Tukey’s multiple comparisons test between three groups, and expressed as mean ± SD. *p < 0.05, **p < 0.01, ***p < 0.001, ns not significant

We determined the role of ISP in hematoma resolution. The gross distribution of ICH was examined on freshly prepared thick brain slices 1, 3, and 7 days after ICH (Fig. 4a). The hematoma volumes were reduced at days 3 and 7, compared to day 1 in both the vehicle-treated mice and ISP-treated mice. However, while ISP administration did not affect hematoma volumes at day 1, it remarkably reduced hematoma volumes at days 3 and 7 after ICH compared to mice in the vehicle group (Fig. 4a, b; day 1: *t* = 0.8342, *p* = 0.4284; day 3: *t* = 2.336, *p* = 0.0477; day 7: *t* = 2.555, *p* = 0.0339).

Macroscopic area of injury for three groups post-ICH were demonstrated at week 4. The images show that the sectional area of the hematoma in the ISP-treated group was much smaller than the vehicle-treated group (Fig. 4c), indicating that ISP administration promotes absorption of hematoma and reduces the severity of this trauma after ICH.

As in our previous studies, ICH occurring in the striatum not only damages the white matter of the brain, but also extends to the contralateral white matter of the spinal cord, such as the dorsal CST (Fig. 4d) [27]. Therefore, we investigated whether ISP treatment decreases white matter disruption both in the brain and associated spinal cord post-ICH. First, we stained the slice of peri-hematoma tissue and found that there was no significant difference between Vehicle and ISP groups (Additional file 4: Fig. S4). When used at an optimal concentration, SMI32 is a monoclonal antibody that recognizes non-phosphorylated neurofilament, which is increased in damaged neurons/axons [37, 38]. The SMI-32/MBP protein ratio is a recognized method for evaluating demyelination [39]. At weeks 4 and 8, vehicle-treated mice revealed a much higher SMI-32/MBP ratio around the hematoma region within the striatum of the brain (Fig. 4e, g; *F* (2,28) = 75.36; 4 W: *p* = 0.0001, 8 W: *p* = 0.0004) and dorsal CST of the cervical spinal cord (Fig. 4f, h; *F* (2,30) = 92.44; 4 W: *p* = 0.0980, 8 W: *p* < 0.0001). Moreover, images exhibited myelin sheath enwrapping axons in ISP group with better integrity in the affected side dorsal CST (Fig. 4f).

**Fig. 5:**
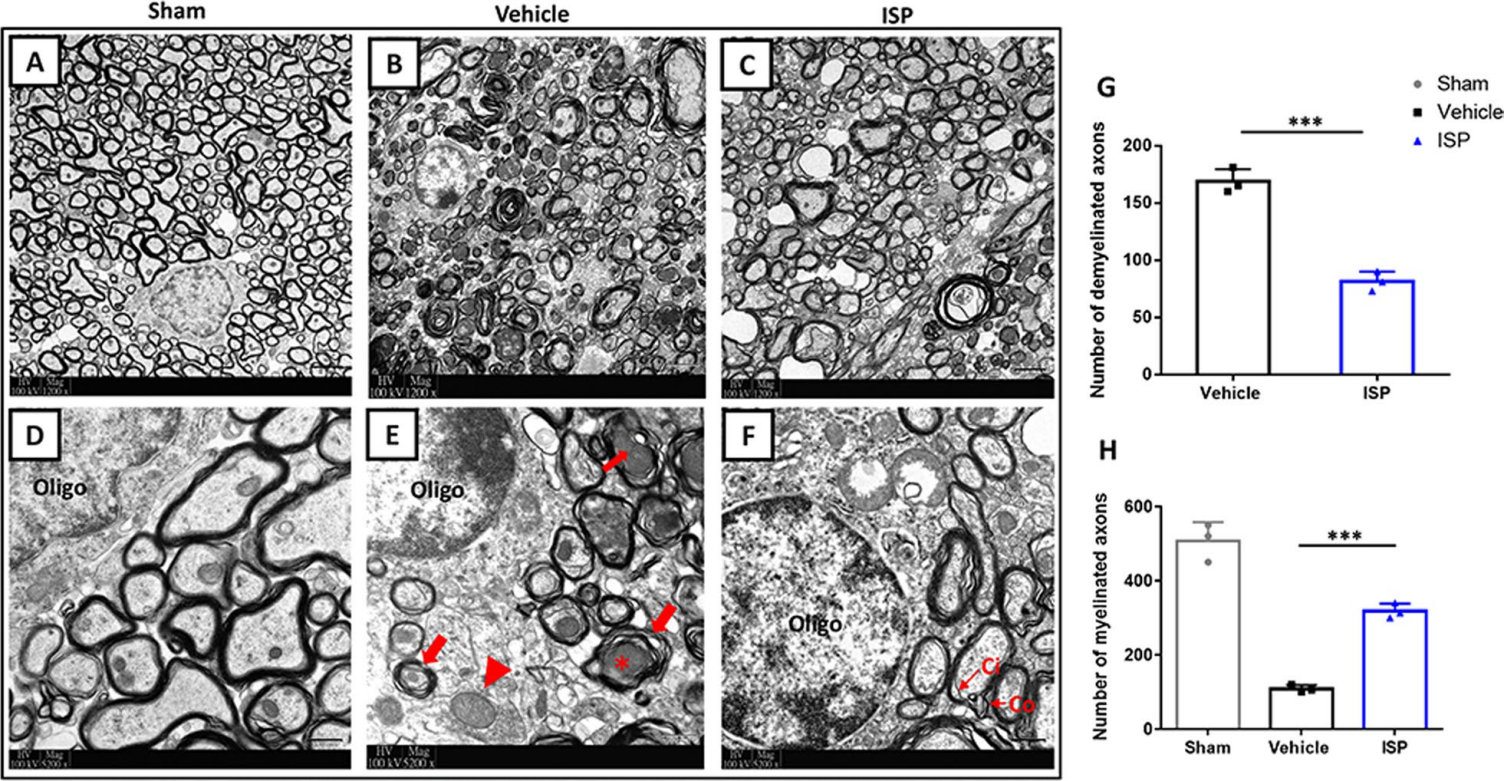
Pathological changes under TEM of the contralateral dorsal CST in the spinal cord at level C5 post-ICH. **a**–**c** Low magnification (1200 × , Scale bar, 2 µm) and **d**–**f** high magnification TEM micrographs (5200 × , Scale bar, 500 nm) showing morphological changes of axon and myelin in Sham, Vehicle, and ISP groups at week 8 post-ICH. **d** Normal axons wrapped by intact myelin sheaths in the Sham group. **e** Swollen mitochondria (red arrowhead) and shrunken, degenerated axons (red asterisks) surrounded by a loose myelin sheath (red arrow). **f** Myelin sheath exhibiting less degeneration after ISP treatment compared with sheaths in the vehicle group. Ci and Co (black arrow) show the inner and outer ends of spiraling cytoplasmic processes of newly formed myelination. **g**, **h** ISP significantly decreased the number of demyelinated axons and increased the number of myelinated axons compared to the vehicle group at week 8. *n* = 3 per group. Data were analyzed by unpaired Student t test between two groups and one-way ANOVA followed by Tukey’s multiple comparisons test between three groups, and expressed as mean ± SD. ***p < 0.001

For the dorsal CST of the spinal cord, we used electron microscopy to demonstrate the morphological details of different groups. Consistent with our previous work, the damaged myelin sheaths were easier to spot due to loose and separated layers or degraded sheath structures in the CST of vehicle-treated mice. In addition, the degenerating axonal changes and edematous mitochondria were found in this group (Fig. 5b, e). In contrast, after 8 weeks of ISP application, the myelin sheaths had much better morphological integrity and the newly formed thinner myelin sheaths were observable (Fig. 5c). We typically observed newly formed myelin sheath morphology in which the outer (Co) and inner (Ci) ends of the axons were enwrapped by oligodendrocytes (Fig. 5f). Consistent with TEM images, the statical analysis confirmed the number of demyelinated axons (*t* = 4; *p* = 0.0004) and myelinated axons in all groups at week 8 post-ICH (Fig. 5g, h; *F* (2, 6) = 113.1, *p* = 0.0005). Taken together with the results of behavioral and electrophysiological functional tests, PTPσ targeting by ISP alleviates cerebral parenchymal damage induced by ICH and promotes axonal and myelin sheaths integrity in the CST of the spinal cord.

## Administration with ISP restores the integrity of neural circuits of the cerebral cortex and spinal cord

Neural circuits from the cerebral cortex and traversing the spinal cord play a vital role in the process of fine motor control [40]. Axons of tracts passing through the internal capsule potentially regulate motor control via neurons located in layer V of the cerebral cortex, including the primary somatosensory cortex (S1) and primary motor cortex (M1) [41]. During behavior, sensory signals are transmitted upward to S1 and motor commands are transmitted downward from M1 to the spinal cord. When ICH occurs in the striatum, the internal capsule is always affected. Here, we investigate how the ISP affects these neural circuits.

**Fig. 6:**
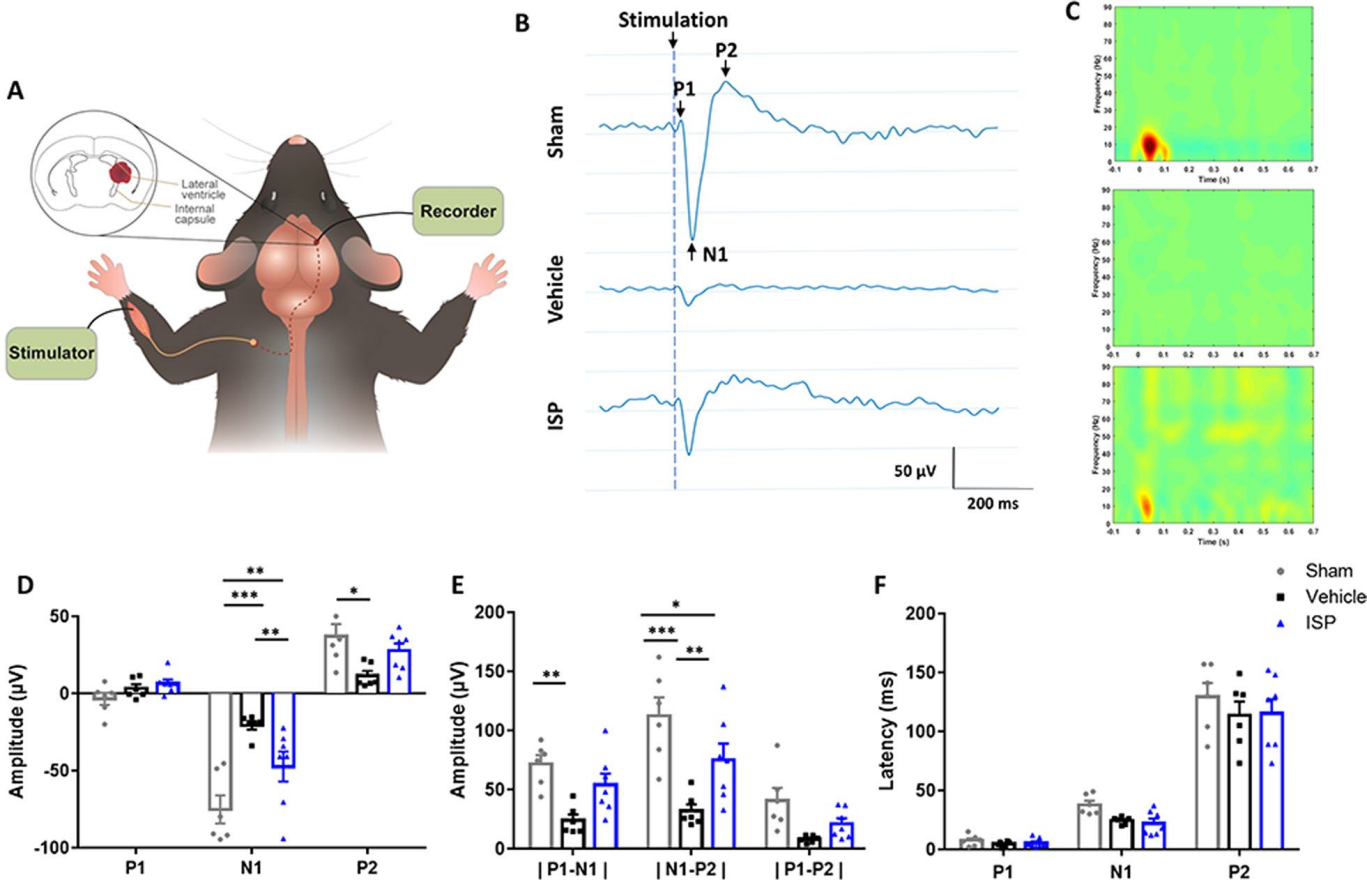
ISP treatment improves nerve conduction as seen from the somatosensory evoked potential (SSEP) analysis at week 8 post-ICH.** a** Location of the stimulator and recording electrodes. **b** Averaged SSEP waveforms of the three groups. The three waveform components are named P1, N1, and P2. **c** Time–frequency analysis of SSEP waveforms. **d**–**f** Comparisons of the three components of SSEP responses, which include the peak amplitude, peak-to-peak amplitudes, and peak latency. The data show that ICH damage decreases the strength of the N1 and P2 components in the vehicle and ISP groups. However, the ISP group still displays a larger amplitude than the vehicle group. No significant difference is observed between the latencies of the P1 and N1 components. However, the latency period of the P2 component is shortened due to the damage. *n* = 6–7 per group. Data were analyzed by one-way ANOVA followed by Tukey’s multiple comparisons test, and expressed as mean ± SD. *p < 0.05, **p < 0.01, ***p < 0.001

The somatosensory evoked potential (SSEP) is an established method for evaluating the integrity of the sensory pathway, where it has been applied to assess white matter injury and functional recovery in animal studies [42]. SSEP elicit and analyze system was built for the assessment of neural circuits of the cerebral cortex and spinal cord after ISP treatment in ICH mice (Additional file 2: Fig. S2). The components of SSEPs, including the P1, N1 and P2, of affected forelimbs in all groups were analyzed at week 8 (Fig. 6a). After ICH, both ISP and vehicle groups displayed significantly altered SSEPs in comparison with the sham group (Fig. 6b). However, the N1 (*F* (2, 48) = 3.253, *p* = 0.0063) and N1-P2 amplitudes (*F* (2, 48) = 23.44, *p* < 0.0001) in the ISP group were significantly increased compared to the vehicle group (Fig. 6d, e). There was no significant difference in latency of P1, N1 and P2 (*F* (2, 48) = 2.181, *p* = 0.1240). The time–frequency responses of SSEP waveforms are also shown (Fig. 6c). From the results, an alpha-band (8–12 Hz) response can be observed around 40 ms after the onset of electrical stimulation for both the sham and ISP groups. In contrast, no notable frequency response can be obtained for the vehicle group. These results indicate that the SSEP response is suppressed due to brain damage and that ISP can partially restore this suppression. In addition, the latency of P2 component in vehicle group was less than the other two groups.

**Fig. 7:**
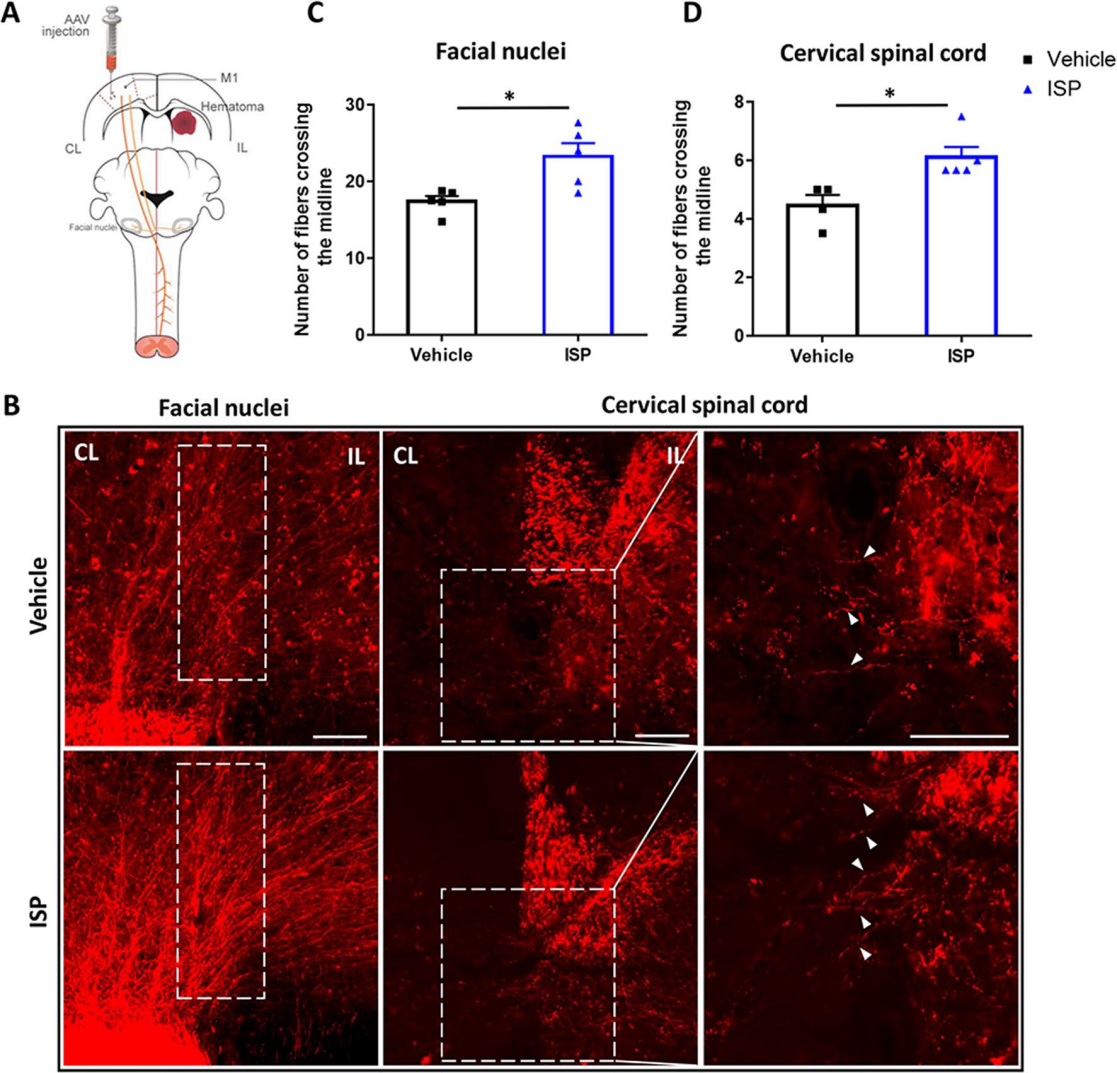
Systemic delivery of ISP increases motor axonal sprouting after ICH. **a** Schematic diagram of CST tracing. The rAAV2/9–CaMKIIa–mCherry vector was injected into the primary motor cortex (M1) at week 6 post-ICH in the vehicle and ISP groups. Two weeks after injection, mice were killed and the brain and spinal cord tissue were harvested. Most fibers from M1 form two tracts: the corticospinal tract (CST), which connects to the spinal cord, and the corticobulbar tract, which connects to the brainstem. The corticobulbar tract and the corticospinal tract project into facial nuclei in the pons and cervical spinal cord of both sides, which is represented in red. **b** Representative images of AAV + fibers (red). Axons crossing the midline, seen in the white boxed area, are within the facial nuclei and cervical spinal cord. The boxed areas in the middle column are enlarged in the right column. Scale bar, 100 µm. **c**, **d** Quantification of axons crossed midline per section from 3 to 5 sections per mice at week 8 post-ICH. n = 3 per group. Data were analyzed by unpaired Student t test, and expressed as mean ± SD. *p < 0.05

Next, to verify the downward motor pathway of the cerebral–spinal neural circuits, which originated from the primary motor cortex, we injected the rAAV2/9–CaMKIIa–mCherry vector into the contralateral primary cortex (M1) (Fig. 7a) to label the major descending corticospinal and corticobulbar tracts, which are substantially involved in movements requiring precise control [41]. Therefore, at week 2 post-rAAV2/9 injection, the brain and cervical spinal cord were harvested, and the number of labeled axons from M1 that crossed the midline into the denervated (ipsilateral) facial nucleus and contralateral spinal cord were quantified to assess the extent of neuroplasticity [43]. As illustrated in our results, mice with ISP treatment showed a marked increase in the number of labeled axons crossing the midline into the ipsilateral side in the facial nuclei (Fig. 7b, c; *t* = 3.109, *p* = 0.0145). Furthermore, at the level of the cervical spinal cord, labeled fibers originating from the contralateral side M1 across the midline into denervated side of grey matter in ISP-treated mice were significantly elevated with respect to the vehicle group (Fig. 7b, d; *t* = 3.216, *p* = 0.0147). Combined with the behavioral results, this indicates that ISP facilitates the restoration of neural circuits of the cerebral cortex and spinal cord by enhancing neuroplasticity after ICH.

### ISP promotes oligodendrocyte myelination through modulation of Erk/CREB pathways in vitro

**Fig. 8:**
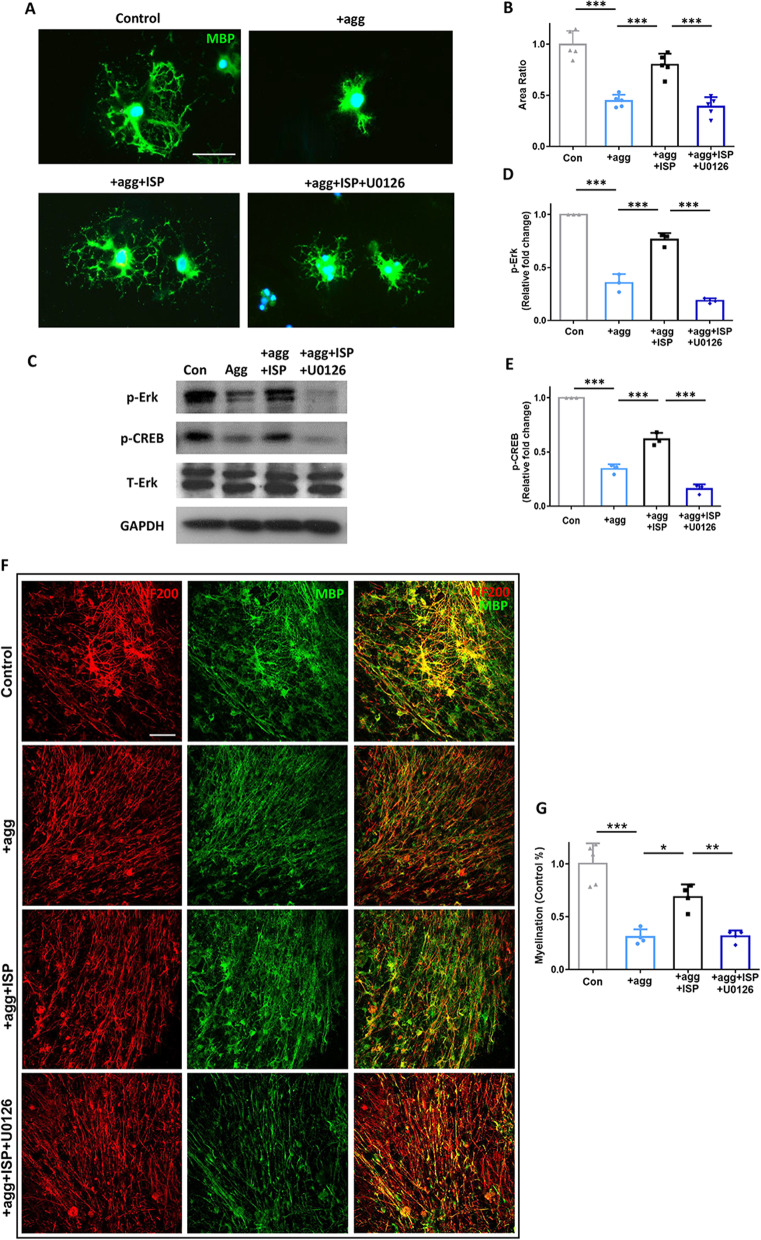
ISP treatment promoted oligodendrocyte outgrowth and myelination through the Erk–CREB signaling pathway. **a** Representative images of oligodendrocytes in the presence of aggrecan in Control (PDL only), Agg (PDL + aggrecan), ISP (aggrecan + ISP), and Erk inhibitor (aggrecan + ISP + U0126) groups as indicated. **b** Statistic showed a significant reduction in oligodendrocyte outgrowth in the aggrecan group relative to the control group, which could be reversed by ISP application. Moreover, inhibition of Erk with U0126 counteracted the elevation caused by ISP treatment. *n* = 5 per group. Scale bar, 50 µm. **c**, **d**, **e** Western blot assay of p-Erk and p-CREB expression in cultured oligodendrocytes of the four groups. Data are normalized to GAPDH expression, *n* = 3 per group. **f**, **g** Representative immunofluorescent images of NF200 (red) and MBP (green) show aggrecan suppressed myelination by oligodendrocytes, which could be reversed by ISP via the Erk signaling pathway, *n* = 8–10 from 4 to 5 independent replicates per group. Scale bar, 100 µm. Data were analyzed by one-way ANOVA followed by Tukey’s multiple comparisons test, and expressed as mean ± SD. *p < 0.05, **p < 0.01, ***p < 0.001

Because myelination increases in mice with ISP treatment as indicated above (Fig. 5), ISP induction was subsequently assessed using a coculture system of DRG and OPC, which is an established in vitro model for evaluating CNS myelination. The outgrowth area of oligodendrocyte is essential during myelination. Our results show that aggrecan inhibited the extent of oligodendrocyte outgrowth, which was reversed by ISP application. Erk/CREB is closely associated with axonal myelination after central nervous injury [44]. To confirm that the effects of ISP on the outgrowth of oligodendrocytes were regulated through the Erk signaling pathway, the Erk inhibitor U0126 was applied to the in vitro assay. Our results showed that U0126 reversed the outgrowth extension which was promoted by ISP treatment (Fig. 8a, b; *F* (3, 16) = 40.19; *p*_Control versus Agg_ < 0.0001, *p*_Agg versus ISP_ = 0.0003, *p*_ISP versus Erk inhibitor_ < 0.0001). In addition, western blotting showed that aggrecan significantly reduced the expression of p-Erk and its downstream p-CREB in oligodendrocytes. Treatment with the ISP limited the aggrecan-induced reduction in p-Erk and p-CREB, which was significantly reversed by U0126 interference (Fig. 8c–e; p-Erk: *F* (3, 8) = 145.2, *p*_Control versus Agg_ < 0.0001, *p*_Agg versus ISP_ < 0.0001, *p*_ISP versus Erk inhibitor_ < 0.0001; p-CREB: *F* (3, 8) = 215.1, *p*_Control versus Agg_ < 0.0001, *p*_Agg versus ISP_ = 0.0003, *p*_ISP versus Erk inhibitor_ < 0.0001).

Next, we utilized a well-established in vitro model of OPCs—DRG co-culture to visualize the remyelination effect of ISP. The myelinated part was identified as co-localizing with MBP + (green) and NF200 + (red) segments in immunostaining. We found that aggrecan prevented significant myelination, which was reversed by ISP application. U0126 was applied to confirm whether ISP regulated oligodendrocyte myelination via the Erk signaling pathway. Our results showed that U0126 significantly reduced the ISP-promoting effect on myelination (Fig. 8f, g; *F* (3, 13) = 30.22; *p*_Control versus Agg_ < 0.0001, *p*_Agg versus ISP_ < 0.0001, *p*_ISP versus Erk inhibitor_ < 0.0001). These in vitro experiments confirm that the ISP promotes oligodendrocytes myelination through modulation of the Erk/CREB pathways.

### ISP treatment alters the protein expression profile within the peri-stroke cortex

**Fig. 9:**
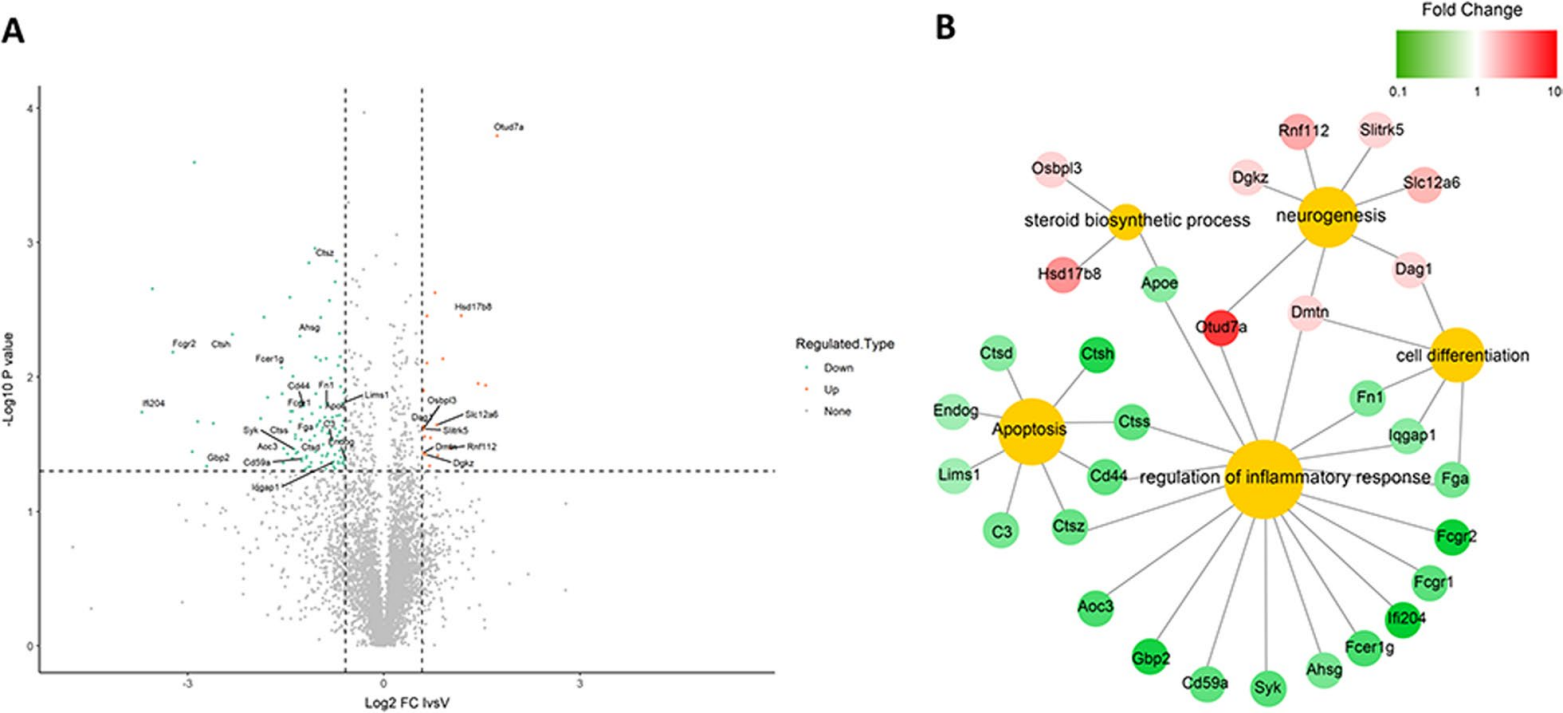
Proteomic analysis of brain tissue around the hematoma zone from animals that were treated with vehicle and ISP post-ICH. **a**, **b** The differential expression of 1.5-fold after ISP administration compared to vehicle administration yielded functional clustering into inflammatory regulation, apoptosis, steroid biosynthesis, and neurogenesis processes. *n* = 3 per group

Given that ISP treatment improved the inflammatory environment, promoted axonal myelination, and accelerated hematoma absorption, we further explored the mechanism of ISP on regulation of the inflammatory response, as well as on neurogenesis and apoptosis. We conducted proteomic analysis on brain tissue around the hematoma zone from animals that had been treated with vehicle or ISP at day 14 post-ICH. Proteomic analysis showed that after ICH, 130 proteins were significantly altered in their expression, typically about 1.5-fold, after ISP treatment when compared with the vehicle group (Fig. 9a). GO analysis suggested that down regulated, pro-inflammation proteins included Ctss, Cd44, Ctsz, Aoc3, Gpb2, Cd59a, Syk, Ahsg, Fcer1g, Lfi204, Fcgr1, Fcgr2, Fga, lqgap1, Fn1, and Apoe (Fig. 9b). Upregulation of pro-neurogenesis related proteins included Otusd7a, Dgkz, Rnf112, Slitrk5, Slc12a6, Dag1, Dmtn, Osbpl3, and Hsd17b8 (Fig. 9b). Down regulated proteins of pro-apoptosis pathways included Ctss, Cd44, Ctsz, C3, Lims1, Endog, and Ctsd (Fig. 9b). Among these, reduced expression of proteins Cd44, Ctsz, C3, and Lims1 may be favorable for nerve regeneration. Further studies are warranted for investigating the functional significance of these proteins after ISP administration for ICH.

## Discussion

We have illustrated that sustained modulation of the proteoglycan receptor PTPσ by intraperitoneal administration of ISP displays white matter–protective and neurorestorative roles following ICH in mice, based on behavioral, morphological, electrophysiological, tract-tracing, and in vitro myelination analyses. In addition, ISP induced a favorable inflammatory environment that may contribute to an enhanced integrity of the white matter. This can be expected to promote the neuroplasticity of brain–spinal circuits and long-term recovery of sensorimotor function.

Neuroinflammation happens immediately after ICH. Therapies that inhibit the injurious phase of microglial activation while augmenting repair would provide great hope for stroke patients [45]. Our results demonstrated that disruption of PTPσ signaling by ISP decreases perihematomal microglia activation. Our findings are consistent with research showing that targeting Toll-like receptor 4 (TLR4) activation affords neuroprotection post-ICH in animal models [46]. Moreover, modulation of microglial phenotype enhances the absorption of hematoma and oedema and also improves white matter integrity and functional recovery post-ICH [47]. Our results, showed increased M2 (anti-inflammation) proteins, signature cytokine IL-10 and Arginase-1 secretion, which may impede inflammatory responses and assist neural protection in CNS disorders [48, 49].

The ICH, in the present study, is adjacent to the internal capsule and striatum, in which fibers of CST are damaged by hematoma and perihematomal edema. This has been shown in some clinical neuroimaging studies [50]. After ICH, the pathological changes in distant regions likely indicate secondary degeneration. Focal inflammation and oxidative stress may lead to persistent degeneration of CST fibers, which have neuron bodies located in the cortex. Hence, the disconnection of CST fibers from cell bodies may closely correlate with long-term prognosis. The long-term data in this study are the first to show that delivered ISP mitigates the lesion of white matter both in the perihematomal brain and implicated spinal dorsal CST at weeks 4 and 8, respectively. Our previous results confirmed the axonal regenerative ability of ISP treatments in spinal ventral root avulsions and dorsal root crush injuries [23, 51]. Nevertheless, whether the prolonged restoration of function is due to the protective effects, or the reparative effects of ISP treatment is not yet known. In this study, besides the in vivo data, the ex vivo assays showed that ISP significantly promoted maturation and myelination of oligodendrocytes through the interference of aggrecan, which is the major component of CSPG. Therefore, the application of ISP demonstrates both protective and reparative effects for myelination after ICH.

Recent studies provide evidence that stroke involves ex situ gliosis and neuronal apoptosis in distant brain regions, such as the substantia nigra and thalamus [52, 53]. As mentioned above, these tract fibers were derived from the corresponding cortex, including the M1 and S1. Hence, we suspect that ICH, a subcortical injury, may affect their cell bodies in the cortex. Thus, further research may focus on the changes in S1 or/and M1, including cortical inflammation and neuronal apoptosis.

In addition, our anatomical tracing illustrated that in the late injury phases post-ICH, ISP promoted corticospinal neuroplasticity, including increased reparatory axonal sprouting across the midline into the contralateral sides both in the facial nuclei and grey matter of the cervical spinal cord. In the present study, unilateral ICH caused modest and transient impairments on the ipsilateral (less-affected) side limbs. Since we only tracked the CST sprouting on the contralateral side, it is unknown whether ISP has the same enhancement effect on ipsilateral CST sprouting. Therefore, we will consider carrying out tracing both CSTs simultaneously afterward.

In addition to morphological evaluation, use of the somatosensory evoked potential (SSEP), as a functional indicator that can measure myelination, enables the exploration of ISP promotion of neurological functional recovery [54]. The reduction in SSEP amplitudes in the vehicle group here may result from a conduction block due to demyelination of CST fibers. Application of ISP to restore the amplitude of SSEP in the long-term was accompanied by improved integrity of myelination, which is consistent with our histological results. In subsequent studies, chronic recording electrodes will be implanted during rehabilitation training to observe dynamic neurological restoration.

Inflammation in situ after ICH leads to secondary tissue damage in the brain and spinal cord [27, 55]. In the present study, we observed significantly less axonal degeneration in ISP-treated animals. Multiple mechanisms could contribute to the weakened neural injury post-ISP administration, including diminished cell apoptosis [55], improved neuroinflammatory environment [35], and increased neurogenesis [12]. These regenerative processes also associate closely with differential protein expression. Thus, we performed proteomic analysis on peri-hematoma of the ISP- and vehicle-treated group. We observed that the genes of inflammation and apoptosis were negatively regulated in neurogenesis and cell differentiation, which is conducive to nerve repair.

As preclinical research, the mode of drug delivery could also be improved. Intraperitoneal injection of ISP is still an invasive drug delivery method. Although it can pass through the BBB to reach the central nervous system (data not shown), the efficiency of the drug through the blood circulation to the lesion remains to be verified. The intranasal route of drug administration is a novel approach for the CNS disorders, which has the advantage of rapid drug delivery to the brain and spinal cord from the nasal mucosa, and also minimizes systemic exposure [56]. Further studies will explore the protected and reparatory effects by intranasal delivery of ISP.

In summary, our findings demonstrate that systemic modulation of PTPσ by ISP improves inflammatory responses, promotes white matter integrity and neuroplasticity in the brain as well as spinal cord, enhancing sensorimotor functions after ICH injury. Moreover, this study demonstrates for the first time that ISP is advantageous in interventions treating ongoing CST degeneration and demyelination, which enables restoration of neurological functions after ICH. In view of the importance of ISP’s reparatory and mitigatory effects on CST, this could be a novel strategy offering encouraging prospects for the clinical treatment of ICH (Additional file 3: Fig. S3, Additional file 4: Fig. S4).

## Conclusions

Our findings reveal compelling evidence that targeting PTPσ by ISP could decrease axonal injury, facilitate WM integrity and long-term neurological recovery after ICH. Manipulation of PTPσ regulates inflammatory environment in the peri-hematomal region, which involve in promoting the integrity of neural circuits in the CNS. Parallel in vitro assays show that PTPσ signaling augment remyelination by activating the Erk/CREB pathway. Thus, our work has shed new light on the pathophysiology of ICH, and ISP may offer substantial promise for its treatment.

## Supplementary Information

**Additional file 1: Fig. S1. ** Affected hindlimb and Less-affected forelimb or hindlimb errors ratio and score on Ladder rung walking after ICH. a b At 1-week timepoint post-ICH, the affected hindlimb of mice in Vehicle and ISP group had similar running score. However, from 3-week post-ICH, both ISP and Vehicle group had progressive restoration. At 8-week timepoint post-ICH, the affected hindlimb of mice in Vehicle group had significant lower scores compared to mice in ISP and Sham. c–f Less-affected forelimb and Less-affected hindlimb on Ladder rung walking are not affected by ICH. Vehicle group and ISP group, n = 9 per group, Sham group, n = 7. All statistical comparisons were made using a two-way repeated measures ANOVA, and a Tukey's multiple comparisons test. Error bars are mean ± SEM.

**Additional file 2: Fig. S2. ** Illustration of the Workflow of neurophysiological data analysis Module. a, b Light microscope images of coronal sections of ISP mice brain at 8-week timepoint. Dotted portion showed hematoma area induced by ICH. Arrows showed the tetrode recording electrode gently lowered through the right-side cortex trajectory. Scale bar: a 500 µm, b 100 µm. c Immunofluorescence images of coronal sections of Sham mice brain at 8-week timepoint. Scale bar: 50 µm. d Neurophysiological data Analysis flowchart, which was based on the MATLAB EEGLAB, including original signals, time–frequency distribution (TFD) and data processing. e, f Averaged data of raw SSEP responses overlay waveform and TFD of 0–90 Hz. g, h Averaged results of filtered SSEP responses.

**Additional file 3: Fig. S3. ** Uncropped blots images of Fig. 3 and Fig. 8 .

**Additional file 4: Fig. S4. ** CSPGs expression around hematoma in Vehicle and ISP groups was showed with fluorescent imaging, respectively. From the images, there was no observable difference between the two groups.

## Data Availability

The data sets used and/or analyzed during the current study are available from the corresponding author on reasonable request.

## Abbreviations

ICH: Intracerebral hemorrhage
CSPGs: Chondroitin sulphate proteoglycans
PTPσ: Protein tyrosine phosphatase-sigma
ISP: Intracellular sigma peptide
WM: White matter
CST: Cortical spinal tract
GAGs: Glycosaminoglycans
OPC: Oligodendrocytes progenitor cell
SCI: Spinal cord injury
MS: Multiple sclerosis
ROS: Reactive oxygen species
IL-1β: Interleukin 1-beta
IL-6: Interleukin 6
TNF: Tumor necrosis factor
IL-4: Interleukin 4
IL-10: Interleukin 10
Arg-1: Arginase-1
SOCS1: Suppressor of cytokine signaling 1
SOCS3: Suppressor of cytokine signaling 3
STAT3: Signal transducer and activator of transcription 3
IL-13: Interleukin 13
ChABC: Chondroitinase ABC
TNF-α: Tumor necrosis factor-alpha
DRG: Dorsal root ganglion neuron
ns: No significance
LAR: Leukocyte common antigen-related
MBP: Myelin basic protein
TEM: Transmission electron microscopy
S1: Primary somatosensory cortex
M1: Primary motor cortex
SSEP: Somatosensory evoked potential
P1: Positive peak 1
P2: Positive peak 2
N1: Negative peak 1
CNS: Central nervous system
agg: Aggrecan
PDL: Poly-D-Lysine
TLR4: Toll-like receptor 4
BBB: Blood–brain barrier
S1FL: Primary somatosensory cortex of forelimb
TFD: Time–frequency distribution
STFT: Short-time Fourier transform
Oligo: Oligodendrocyte
GO: Gene ontology
