# Supplementary figures and images for: Modulation of the proteoglycan receptor PTPσ promotes white matter integrity and functional recovery after intracerebral hemorrhage stroke in mice
Source: J Neuroinflammation. 2022 Aug 18;19:207. doi: 10.1186/s12974-022-02561-4 (PMC9387079; doi:10.1186/s12974-022-02561-4)

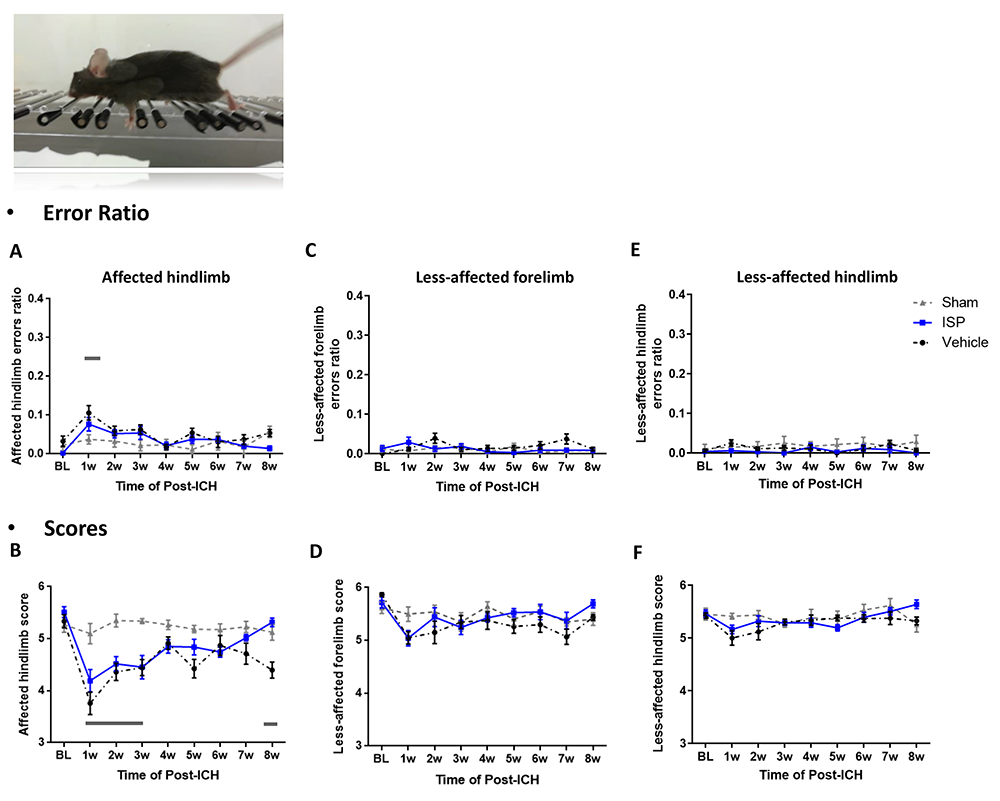

Supplement: Supplementary file 1 — Additional file 1: Fig. S1. Affected hindlimb and Less-affected forelimb or hindlimb errors ratio and score on Ladder rung walking after ICH. a b At 1-week timepoint post-ICH, the affected hindlimb of mice in Vehicle and ISP group had similar running score. However, from 3-week post-ICH, both ISP and Vehicle group had progressive restoration. At 8-week timepoint post-ICH, the affected hindlimb of mice in Vehicle group had significant lower scores compared to mice in ISP and Sham. c–f Less-affected forelimb and Less-affected hindlimb on Ladder rung walking are not affected by ICH. Vehicle group and ISP group, n = 9 per group, Sham group, n = 7. All statistical comparisons were made using a two-way repeated measures ANOVA, and a Tukey's multiple comparisons test. Error bars are mean ± SEM. [file 12974_2022_2561_MOESM1_ESM.tif]

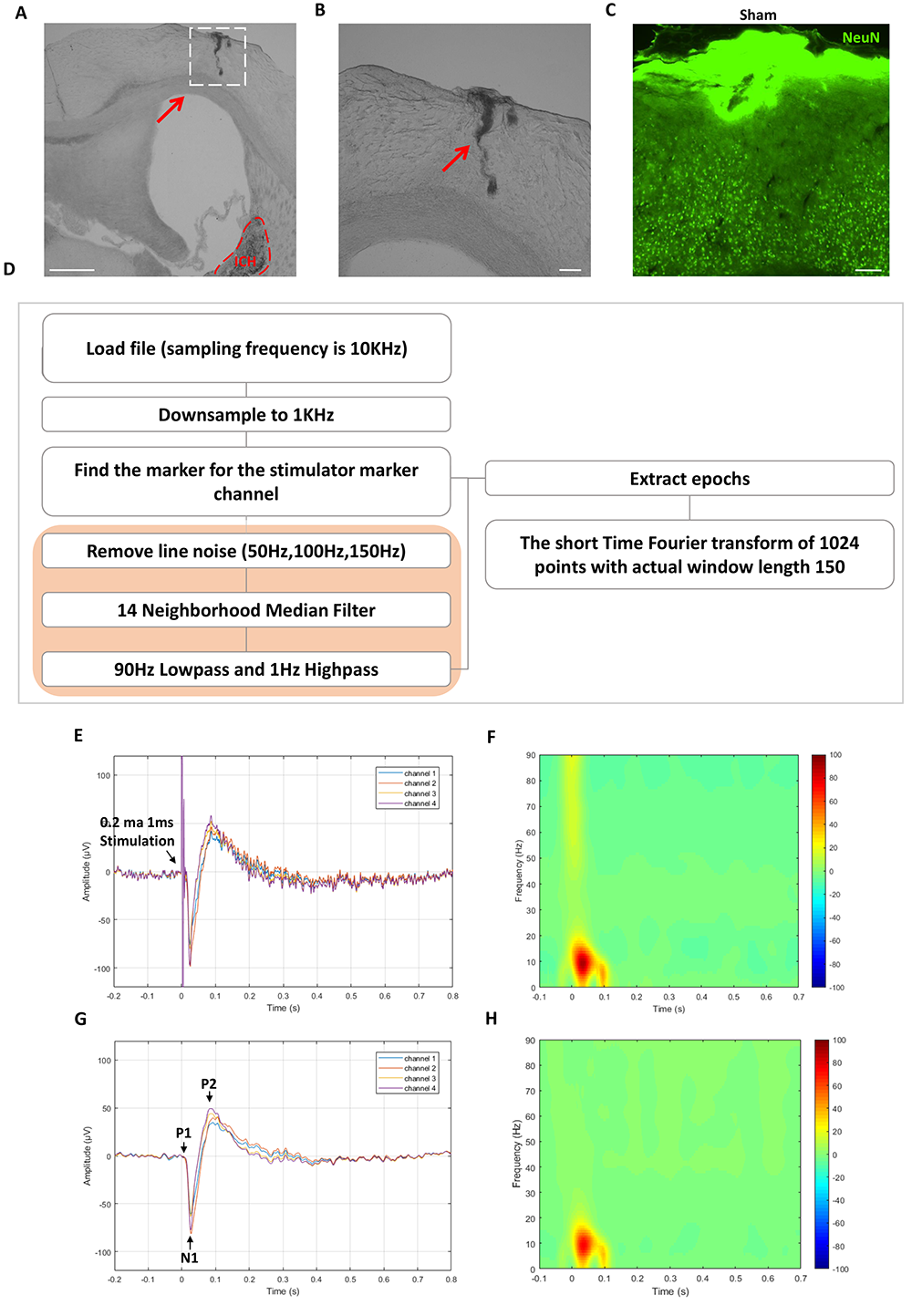

Supplement: Supplementary file 2 — Additional file 2: Fig. S2. Illustration of the Workflow of neurophysiological data analysis Module. a, b Light microscope images of coronal sections of ISP mice brain at 8-week timepoint. Dotted portion showed hematoma area induced by ICH. Arrows showed the tetrode recording electrode gently lowered through the right-side cortex trajectory. Scale bar: a 500 µm, b 100 µm. c Immunofluorescence images of coronal sections of Sham mice brain at 8-week timepoint. Scale bar: 50 µm. d Neurophysiological data Analysis flowchart, which was based on the MATLAB EEGLAB, including original signals, time–frequency distribution (TFD) and data processing. e, f Averaged data of raw SSEP responses overlay waveform and TFD of 0–90 Hz. g, h Averaged results of filtered SSEP responses. [file 12974_2022_2561_MOESM2_ESM.tif]

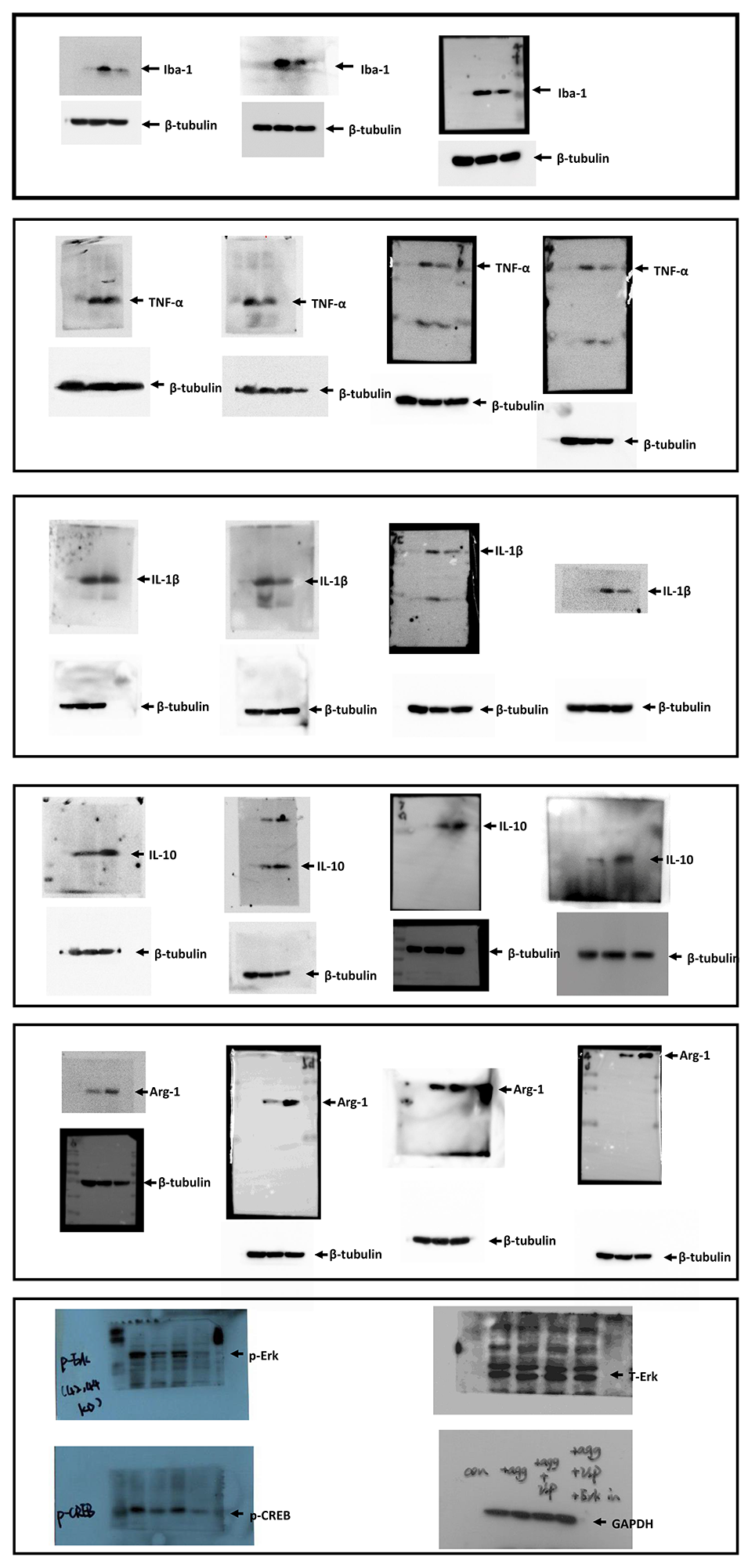

Supplement: Supplementary file 3 — Additional file 3: Fig. S3. Uncropped blots images of Fig. 3 and Fig. 8 . [file 12974_2022_2561_MOESM3_ESM.tif]

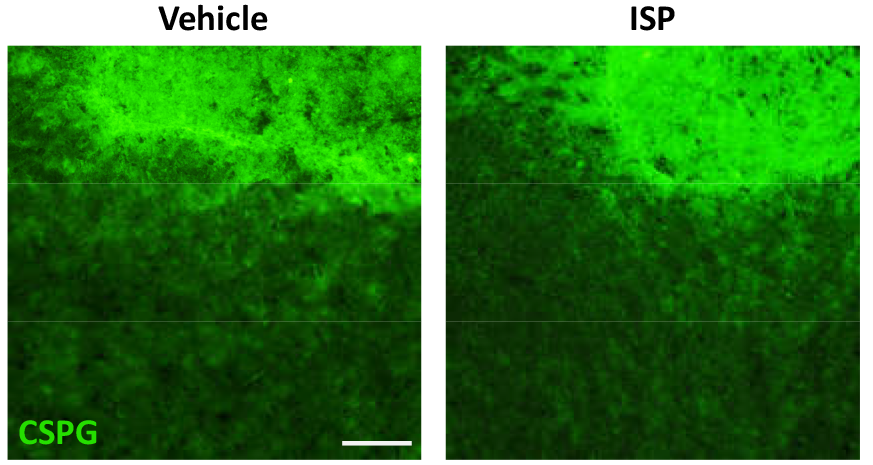

Supplement: Supplementary file 4 — Additional file 4: Fig. S4. CSPGs expression around hematoma in Vehicle and ISP groups was showed with fluorescent imaging, respectively. From the images, there was no observable difference between the two groups. [file 12974_2022_2561_MOESM4_ESM.tif]
